# Disparities and Outcomes in the First and Second Year of the Pandemic on Events of Acute Myocardial Infarction in Coronavirus Disease 2019 Patients

**DOI:** 10.3390/medicina60040597

**Published:** 2024-04-04

**Authors:** Jasninder Singh Dhaliwal, Manraj S. Sekhon, Arush Rajotia, Ashujot K. Dang, Prabh Partap Singh, Maham Bilal, Hemamalini Sakthivel, Raheel Ahmed, Renuka Verma, Kamleshun Ramphul, Prabhdeep S. Sethi

**Affiliations:** 1 Department of Internal Medicine, University of California Riverside School of Medicine, Riverside, CA 92521, USA; 2 School of Medicine, University of California Riverside School of Medicine, Riverside, CA 92521, USA; 3 Department of Internal Medicine, Dow University of Health Sciences, Karachi 74200, Pakistan; 4 One Brooklyn Health System/Interfaith Medical Ctr Program, Brooklyn, NY 11213, USA; 5 Royal Brompton Hospital, Part of Guy’s and St. Thomas’ NHS Foundation Trust, London SW3 6NP, UK; 6 Department of Internal Medicine, Kirk Kerkorian School of Medicine at UNLV, Las Vegas, NV 89154, USA; 7 Independent Researcher, Triolet 21504, Mauritius; adramphul@hotmail.com

**Keywords:** COVID-19, cardiovascular complications, national inpatient sample, United States, mortality, epidemiology

## Abstract

*Background and Objectives*: Coronavirus disease 2019 (COVID-19) caused several cardiovascular complications, including acute myocardial infarction (AMI), in infected patients. This study aims to understand the overall trends of AMI among COVID-19 patients during the first two years of the pandemic and the disparities and outcomes between the first and second years. *Materials and Methods*: The retrospective analysis was conducted via the 2020 and 2021 National Inpatient Sample (NIS) database for hospitalizations between April 2020 and December 2021 being analyzed for adults with a primary diagnosis of COVID-19 who experienced events of AMI. A comparison of month-to-month events of AMI and mortality of AMI patients with concomitant COVID-19 was made alongside their respective patient characteristics. *Results*: Out of 2,541,992 COVID-19 hospitalized patients, 3.55% experienced AMI. The highest rate of AMI was in December 2021 (4.35%). No statistical differences in trends of AMI mortality were noted over the 21 months. AMI cases in 2021 had higher odds of undergoing PCI (aOR 1.627, *p* < 0.01). They experienced higher risks of acute kidney injury (aOR 1.078, *p* < 0.01), acute ischemic stroke (aOR 1.215, *p* < 0.01), cardiac arrest (aOR 1.106, *p* < 0.01), need for mechanical ventilation (aOR 1.133, *p* < 0.01), and all-cause mortality (aOR 1.032, 95% CI 1.001–1.064, *p* = 0.043). *Conclusions*: The incidence of AMI among COVID-19 patients fluctuated over the 21 months of this study, with a peak in December 2021. COVID-19 patients reporting AMI in 2021 experienced higher overall odds of multiple complications, which could relate to the exhaustive burden of the pandemic in 2021 on healthcare, the changing impact of the virus variants, and the hesitancy of infected patients to seek care.

## 1. Introduction

Since the initial outbreak of Severe Acute Respiratory Syndrome Coronavirus 2 (SARS-CoV-2) in late December 2019, more than 703 million people have been infected with coronavirus disease 2019 (COVID-19), which has led to an estimated 6.9 million deaths [1,2]. The rapid impact of COVID-19 on millions of individuals prompted the World Health Organization (WHO) to declare it a pandemic in March 2020 [3]. Although the major focus is on pulmonary consequences, COVID-19 is correlated with significant direct and indirect cardiovascular implications, with the latter likely holding greater significance, specifically in time-sensitive cardiovascular emergencies [4]. These cardiovascular complications contribute significantly to disease mortality [3]. COVID-19 interacts with the cardiovascular system through various mechanisms, leading to complications such as myocardial injury and dysfunction in individuals with underlying cardiovascular diseases [3]. One implicated mechanism involves the role of angiotensin-converting enzyme2 (ACE2) as a receptor for coronavirus, with its high expression mainly observed in the heart and lungs [5].

A study conducted by Katsoularis et al. involving approximately 86,742 COVID-19 patients reveals a significant risk of individuals developing acute myocardial infarction as a consequence of COVID-19 [6]. This underscores the vast clinical picture of COVID-19, emphasizing the importance of vaccination against the virus. The research indicates that the risk of incidence of AMI among COVID-19 ranges from 1.1% to 8.9% [6]. Prior research has suggested the possible mechanism of AMI (Type 1) in respiratory infections, which includes the development of pro-inflammatory phase, which can trigger the destabilization of coronary atherosclerotic plaque [5]. Another type of myocardial infarction (Type 2) that does not involve obstructive coronary arteries could be triggered by hypoxia, hypotension and tachycardia, which are commonly observed in acute respiratory failure [5,6]. In addition, the virus can have several direct and indirect impacts on cardiac myocytes, which can trigger multiple varied cardiovascular complications, such as cardiac arrhythmias [7], heart failure [8], and myocarditis [9]. Myocardial injury can present with various electrocardiographic expressions, ranging from normal EKG to ST-elevated acute myocardial infarction (5). COVID-19-positive patients with pre-existing comorbidities such as hypertension, prior cardiovascular disease, and diabetes have also been predisposed to a higher risk of cardiovascular complications and mortality [10]. Post-COVID syndrome, as evidenced by many studies, has been associated with a multitude of cardiovascular risks among affected individuals. These risks may include arrhythmias, thromboembolic events and myocardial infarction [11,12]. Therefore, comprehensive monitoring and management of cardiovascular health in individuals recovering from COVID-19 are imperative to mitigate the long-term sequelae of the disease. Over the course of the pandemic, many drastic measures and changes were brought forward to reduce the impact of the virus, such as lockdown protocols [13] and vaccinations [14].

Several past studies using the National Inpatient Sample (NIS) database have evaluated different aspects of how the virus influenced the odds and outcomes of AMI patients in 2020 [15,16,17,18]. These studies have offered valuable insights into the intersection of COVID-19 and cardiovascular health, underscoring the increased risks and challenges encountered by individuals affected by both conditions. However, there is currently a gap in the literature regarding comparisons of outcomes for COVID-19 patients with AMI between the two years of the pandemic. Therefore, we propose a retrospective study to fill this void and aim to improve our understanding of potential differences in outcomes over time, thus informing healthcare policies more effectively.

## 2. Materials and Methods

### 2.1. Data Source

We evaluated patients with a primary diagnosis code for COVID-19 in 2020 and 2021 via the NIS. The NIS is produced each year under the supervision of the Healthcare Cost Utilization Project (HCUP) and the Agency for Healthcare Research and Quality (AHRQ), and is a member of the plethora of databases released by the HCUP which also includes Kids’ Inpatient Database (KID), Nationwide Ambulatory Surgery Sample (NASS), Nationwide Emergency Department Sample (NEDS), Nationwide Readmissions Database (NRD), State Inpatient Databases (SID), State Ambulatory Surgery and Services Databases (SASD), and State Emergency Department Databases (SEDD). It is the biggest well-established all-payer publicly accessible database which contains de-identified data from around 20% of hospital records across 47 states and the District of Columbia in the United States. The use of the discharge code “DISCWT” allows users to estimate more than 97% of the population. On average, each year’s database contains at least 7 million patients in unweighted form, and 35 million hospitalizations nationally [19,20].

The 2021 NIS was released in 2024 and contains, to date, the most recent national data covering COVID-19 hospitalizations by the HCUP. The NIS contains multiple patient records at a de-identified level, along with up to 40 clinical diagnoses and 25 procedures that can be evaluated using the International Classification of Diseases (ICD) codes. For the NIS 2020 and 2021, users are required to use ICD-10 codes. Various patient demographics are also included in the databases along with details on hospital characteristics such as location/teaching status, region, and bed size [19].

### 2.2. Sample and Statistical Analysis

The ICD-10 diagnosis code for COVID-19, “U071”, was started on 1 April 2020, and as per the HCUP’s recommendations, we only retained patients from 1 April 2020 to 31 December 2021 for this study [19].

First, all cases of COVID-19 with a primary diagnosis were extracted. We excluded those of ages < 18 years. Cells missing data for month or year were excluded from this study. The ICD-10 codes “I2101”, “I2102”, “I2109”, “I2111”, “I2119”, “I2121”, “I2129”, “I213”, “I214”, “I219”, “I21A1”, and “I21A9” were used to identify patients with AMI [21]. The month-to-month event of AMI among COVID-19 hospitalizations was evaluated using linear-by-linear association.

We then proceeded to retain only COVID-19 cases with a coexisting code for AMI, and two groups were made: patients admitted in 2020 vs. those admitted in 2021. Additional comorbidities, diagnoses, and procedures were included via their ICD-10 codes [20,22,23,24,25,26,27,28,29,30]. We also used Glasheen et al.’s version of the Charlson Comorbidity Index (CCI) scoring to evaluate the burden of comorbid disease in our study [30]. Cells missing data for a month or year were excluded from this study. The characteristics of the two groups were compared using chi-square tests for categorical variables (weekend admissions, sex, primary payer, race, median household income, hospital bed size, teaching status, region, and comorbidities) (reported as frequency (%)) and *t*-tests for continuous variables (age, CCI score, length of stay, and hospital charge) (reported as mean ± SD) [19].

**Figure 1:**
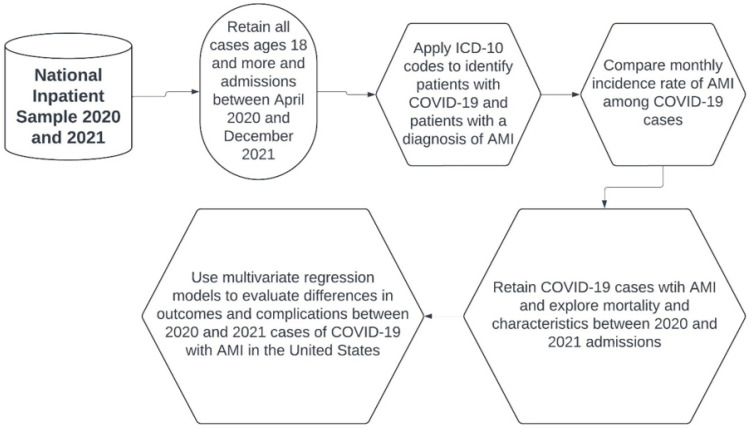
Flow chart of the selection and analysis of our study.

The primary outcome was all-cause mortality among COVID-19 patients with AMI. We further explored the use of coronary artery bypass graft surgery (CABG), percutaneous coronary intervention (PCI), mechanical ventilation, intra-aortic balloon pump (IABP), and events of acute kidney injury (AKI), cardiogenic shock, cardiac arrest, and cardiac arrhythmias (atrial fibrillation, ventricular fibrillation, and supraventricular tachycardia) in these patients. The mean length of stay (LOS) and their mean hospital charges were also compared. The complications and outcomes were evaluated via multivariable regression analyses as an adjusted odds ratio (aOR) along with their 95% confidence interval (95% CI) and *p*-value. We retained statistical significance for *p* < 0.05 throughout the study. The analyses were performed using SPSS 29.0 (IBM Corp., Armonk, NY, USA) and STATA 18.0 MP (StataCorp LLC, College Station, TX, USA) (Figure 1).

As the HCUP provided the data in de-identified form, the study was exempted from any ethical or IRB approval. We also adhered strictly to the guidelines and rules of the HCUP in the use of these databases. All cases < 11 and any individual cell that involved data from less than two hospitals were not included in the results. Furthermore, the authors have not made any attempt to identify any patients or hospitals during the analysis. All authors who had access to the original database signed the Data Use Agreement and underwent the mandatory training. The database cannot be shared. Anyone wishing to obtain access to the NIS are required to contact the HCUP [19].

## 3. Results

In the United States, an estimated 2,541,992 adults with COVID-19 were hospitalized between April 2020 and December 2021. This consisted of 1,019,860 admissions between April and December 2020 followed by an additional 1,522,131 cases between January and December 2021.

### 3.1. AMI Trend among COVID-19 Cases

**Figure 2:**
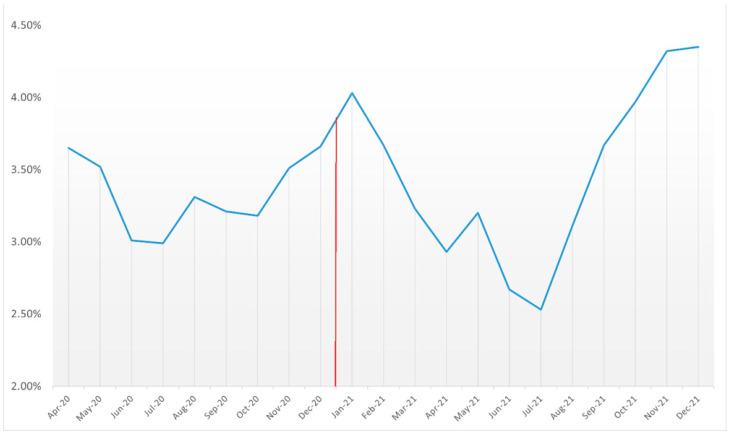
AMI events among adult COVID-19 cases between April 2020 and December 2021 (red line denotes the FDA approval of COVID-19 vaccines).

Throughout the 21 months of our analysis, the incidence of AMI among individuals diagnosed with COVID-19 fluctuated. Initially, between the months of April and July 2020, a decline in incidence was noted from 3.65% to 2.99%. However, a subsequent rise was seen leading up to January 2021 as it reached 4.03%, following which, another drop was noted until July 2021, where the incidence was 2.53%. This decline was followed by a sharp rise until December 2021, where 4.35% of patients with COVID-19 reported AMI. The variation in the incidence of AMI was statistically significant with a p_trend_ < 0.01. Across the 21 months of our study, the overall rate of AMI among COVID-19 patients was estimated at 3.55% (Table 1, Figure 2).

### 3.2. Comparing AMI Cases in COVID-19 Patients in 2020 vs. 2021

Whilst evaluating 90,180 AMI cases among COVID-19 patients, 34,735 were in the 2020 cohort and 55,445 were in 2021.

### 3.3. Baseline Characteristics

AMI cases in COVID-19 patients were younger in 2021 as compared to 2020, with a mean age of 70.31 vs. 72.38 years (*p* < 0.01) (Table 2).

There was no significant difference in weekend admission rates between 2020 and 2021, with both groups predominantly involving males; however, there was a slightly higher presence of females in 2021 compared to 2020 (40.5% vs. 39.3%, *p* < 0.01). Additionally, there was a notable shift in primary payer demographics, with a decrease in patients covered by Medicare: 69.9% of cases in 2020 vs. 66.1% cases in 2021 (*p* < 0.01). Conversely, there was increase in patients with private insurance from 15.5% in 2020 to 17.8% in 2021 (*p* < 0.01). Racially, patients of White ancestry formed the biggest part of the AMI cases, accounting for 55.6% in 2020, which notably increased to 65.0% in 2021 (*p* < 0.01). Conversely, there was a considerable decrease in patients of Hispanic ancestry from 18.0% in 2020 to 12.4% in 2021, along with a slight decline in the percentage of patients of Black ancestry, from 17.8% in 2020 to 16.1% in 2021. (*p* < 0.01). An analysis of median household income distribution revealed that most cases were in the 0–25th household income percentile in both groups (36.8% in 2020 and 36.8% in 2021, *p* < 0.01). Furthermore, patients were predominantly treated in large bed-size hospitals (46.6% in 2020 and 44.6% in 2021) and hospitals classified as urban teaching centers (71.2% in 2020, 68.3% in 2021, *p* < 0.01). Geographically, there was a notable concentration of AMI cases in the southern regions of the US (38.7% in 2020, 42.8% in 2021, *p* < 0.01).

It was found that cases in 2020 had higher prevalences of hypothyroidism (14.1% vs. 13.2%, *p* < 0.01), dyslipidemia (53.2% vs. 49.4%, *p* < 0.01), diabetes (49.0% vs. 45.9%, *p* < 0.01), chronic kidney disease (40.5% vs. 39.2%, *p* < 0.01), prior CABG (8.4% vs. 7.6%, *p* < 0.01), prior PCI (10.7% vs. 9.2%, *p* < 0.01), family history of CAD (4.5% vs. 4.0%, *p* < 0.01), prior stroke (8.3% vs. 7.6%, *p* < 0.01), and prior myocardial infarction (9.4% vs. 8.8%, *p* < 0.01). On the contrary, cases in 2020 had fewer smokers (28.1% vs. 30.1%, *p* < 0.01), cases with liver cirrhosis (5.2% vs. 5.8%, *p* < 0.01), alcohol abuse (1.8% vs. 2.4%, *p* < 0.01), obesity (20.4% vs. 25.5%, *p* < 0.01), drug abuse (2.0% vs. 2.8%, *p* < 0.01), and COPD (20.7% vs. 21.9%, *p* < 0.01).

The 2020 cohort exhibited a higher overall mean Charlson Comorbidity Index (CCI) score (5.04 vs. 4.91. *p* < 0.01). Patients admitted in 2021 had a longer mean length of stay compared to the 2020 cohort (11.20 days vs. 9.95 days, *p* < 0.01), with a higher mean hospital charge (USD 155,587 vs. USD 130,538, *p* < 0.01).

### 3.4. Trends in Mortality

**Figure 3:**
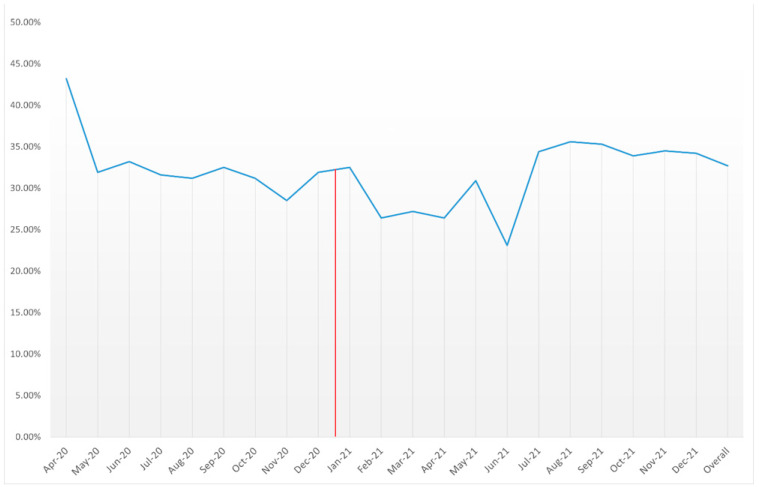
Mortality rates among COVID-19 cases with AMI between April 2020 and December 2021 (red line denotes FDA approval of COVID-19 vaccines).

The overall mortality rate fluctuated between the 21 months of the study. The initial mortality rates in April 2020 were relatively high at 43.20%, gradually decreasing over subsequent months before reaching a low of 23.1% in June 2021. However, from July 2021 onwards, mortality rates experienced an increase, peaking at 35.6% in August 2021. Despite these fluctuations, statistical significance was not observed across the study period (p_trend_ = 0.103), averaging at 32.70% over the study period (Table 3, Figure 3).

### 3.5. Events of Cardiac Arrhythmias

Multivariable regression models for cardiac arrhythmias found that in 2021, there were higher odds of events of ventricular tachycardia (aOR1.126, 95% CI 1.062–1.194, *p* < 0.01) among COVID-19-positive patients who experienced AMI than those in 2020. No differences were seen for atrial fibrillation or supraventricular tachycardia (Table 4).

### 3.6. Complications and Outcomes

Several differences in outcomes between the two years of the pandemic were demonstrated. In 2021, patients with AMI whilst admitted for COVID-19 had a higher likelihood of having a PCI (aOR 1.627, 95% CI 1.454–1.822, *p* < 0.01), while also reporting more complications such as acute kidney injury (aOR 1.078, 95% CI 1.047–1.110, *p* < 0.01), acute ischemic stroke (aOR 1.215, 95% CI 1.113–1.328, *p* < 0.01), events of cardiac arrest (aOR 1.106, 95% CI 1.050–1.166, *p* < 0.01), the need for mechanical ventilation (aOR 1.133, 95% CI 1.096–1.172, *p* < 0.01), and all-cause mortality (aOR 1.032, 95% CI 1.001–1.064, *p* = 0.043). There was no statistically significant result for events of cardiogenic shock and the need for IABP between these two years (Table 3).

## 4. Discussion

This study has brought to light several key differences in the incidences of AMI among COVID-19 patients, patient characteristics, and outcomes of the AMI cases between the first two years of the pandemic in the United States.

We found that AMI events among COVID-19 cases reached an initial peak during January 2021. This corresponds with another peak previously reported regarding the daily deaths during that same month, which to date was the highest number of COVID-related deaths reported in the United States and worldwide [31,32]. The analysis also confirmed a continuous drop in AMI cases between January and July 2021, which could also correlate with the introduction of vaccination access to the general public and expansion of vaccination campaigns in the United States. However, we further noticed that from July 2021, the incidence of AMI among COVID-19 patients rose constantly until the end of our study (December 2021). These fluctuations underscore an intricate interaction between COVID-19 and cardiovascular conditions, suggesting possible periods of increased vulnerability. This may be linked to the waning levels of antibodies following vaccination, the changes in preventive protocols to prevent infection in at-risk groups, and the impact of viral mutations (such as Delta, Omicron, Alpha, Gamma, and Beta) and their pathophysiological impact on the cardiovascular system [33,34,35,36,37,38,39,40]. Moreover, it is crucial to delve into further studies exploring the hesitancy of patients with AMI symptoms to seek medical care due to fear of being infected with COVID-19 while being hospitalized [41,42]. Understanding these factors is essential for formulating approaches to encourage prompt medical attention and mitigate negative influence on cardiovascular health.

The average mortality rate among COVID-19 patients with AMI (32.70%) is higher than the mortality rate in AMI patients without COVID-19 (4.9–7%) [43], as reported in previous studies. This correlates with other studies among COVID-19 cases who experienced AMI. In a study by Chan et al. investigating AMI cases among COVID-19 patients in California, an estimated 43.2% of their patients did not survive [44]. A systematic review by Rus et al. suggested that mortality can reach 76% [5]. This higher mortality rate is linked to the impact of the virus on the patients, with multi-systemic insults that predispose them to poorer outcomes. Moreover, during the pandemic, the diagnosis, availability, and timely treatment of AMI cases may have also been impacted. Encouraging individual hospital-based retrospective studies will help us to understand the main shortcomings and allow the healthcare system to adjust and prepare for the future. Similarly, further investigations are imperative to examine the roles of cardiac complications such as myocarditis and embolus leading to AMI in COVID-19 patients with normal coronary arteries.

In this cohort, patients of White ancestry comprised the largest proportion of AMI cases in both years, significantly surpassing the representation of individuals of Black and Hispanic ancestry. Prior research has suggested that individuals of Black and Hispanic ancestry might experience delayed diagnosis due to implicit and explicit bias, as well as disparities in health care access and management. The current study findings demonstrate the crucial need to tackle the well-established racial and ethnic gaps in cardiovascular disease outcomes [45]. A systemic review of 16 studies revealed that a diverse workforce with more minority physicians could lead to improved health outcomes among marginalized populations like Black and Hispanic ancestry communities [46]. Additionally, males constitute the majority of the population in this current study. This finding is in line with the study of Kumar et al., which similarly demonstrated a higher prevalence of AMI in males infected with COVID-19 [47]. This may be due to gender disparity in cardiovascular care, where females remain underdiagnosed and undertreated in comparison to their male counterparts [48,49]. Another possible explanation of male predominance can lie in the pathophysiological differences between males and females, with females typically exhibiting a lesser extent of arterial plaque buildup, known as atherosclerotic burden, compared to men during a myocardial infarction (MI) [50]. Elevated microvascular resistance has been associated with unrecognized myocardial infarction (MI) in women, and after menopause, women are more likely to experience higher rates of resistance in their coronary microvasculature. These alterations in the microvasculature due to aging and menopause might contribute to women experiencing more unusual symptoms that often remain undetected [50]. These variations between males and females warrant further research to better understand their potential implications for clinical practice, ultimately aiming to optimize cardiovascular care for individuals of all genders. Furthermore, this study underscores the prominence of southern regions as major contributors to the overall prevalence of AMI. Multiple factors, including the prevalence of cardiovascular risk factors, low level of education, unhealthy dietary habits, lack of exercise, and inadequate access to medical services, may contribute to this geographic concentration [51]. Comprehending the core etiologies of an increased prevalence of AMI in the southern regions is crucial for devising strategies and public health interventions with the purpose of alleviating the cardiovascular complications and improving health outcomes in these areas. This study also demonstrates a decrease in patients covered by Medicare, while an increase in the proportion of patients with private insurance was noted. This shift emphasizes changes in the patterns of healthcare coverage throughout the course of the pandemic. This makes it crucial for healthcare providers and policy makers to better allocate resources and design reimbursement systems which can efficiently fulfil the needs of patients with AMI, while also ensuring equitable access to medical care [52].

There were also some discrepancies in the patient demographics between the AMI patients in our two groups. AMI patients were younger in 2021, with varying differences in several comorbidities between the two groups, and expressed a lower mean CCI score in 2021 as compared to 2020. Based on the data provided by the CDC, the mortality rates of all COVID-19 cases among various younger adult groups also rose in 2021; for example, the mortality among COVID-19 patients of ages 35–44 and 45–54 rose from 16.0 and 45.2 per 100,000 standard population to 40.6 and 97.9 per 100,000 standard population in 2021, while the rise in much older groups was less drastic, from 644.4 to 649.3 among patients aged 75–84 [53]. There are several potential reasons for this finding. Variants of the virus, notably Delta and Omicron, which were predominant during the 2021 outbreak, exhibited higher transmissibility, resulting in more widespread infections among younger individuals [54]. Additionally, greater social interaction among younger age groups may have contributed to increased exposure to the virus. Furthermore, vaccination hesitancy among the younger population may have predisposed them to worse outcomes following COVID-19 [55]. We therefore encourage additional retrospective studies to identify the factors associated with the differences seen, as it can be utilized as a model to improve the outcomes in any future pandemics [56]. Efforts to address these factors, including vaccination and public health measures, may alleviate the impact of the pandemic on all age groups.

Finally, our study also found several differences in the outcomes between the 2021 and 2020 AMI cases in COVID-19 patients. The CDC confirmed that there was a higher mortality rate among COVID-19 patients in 2021, as the overall deaths rose from 93.2 per 100,000 individuals to 111.4 per 100,000 individuals. A similar rise was seen as 2021 AMI cases reported higher odds of death in our study [53]. Kumar et al. suggested that high-risk patients and those with multiple comorbidities should undergo an ECG on admission, along with cardiac enzyme testing. Implementing such protocols may ultimately mitigate the mortality rate and reduce the length of hospital stay [47]. Furthermore, the current study found that AMI patients admitted in 2021 for COVID-19 have a higher likelihood of undergoing PCI. The higher use of PCI in 2021 could correlate with the improvement in the access of care and distribution of resources as compared to the first year of the pandemic [57,58]. However, despite this increase in procedures, they experienced more complications compared to previous years, including acute kidney injury, acute ischemic stroke, cardiac arrest events, the need for mechanical ventilation, and an increased risk of all-cause mortality. The possible explanation of this finding lies in the fact that vaccine efficacy was compromised when Delta and Alpha became the dominant variants of COVID 19 in 2021. Studies have shown that these variants were associated with a reduced protection effect on COVID-19-related mortality, suggesting higher pathogenicity of the emerging variant (Delta) or suboptimal reaction to vaccination and declining immunity over time [59,60].

Our study had several strengths, most notably the access to up-to-date nationwide health data from more than 80,000 COVID-19-infected individuals diagnosed with AMI. The dataset from the National Inpatient Sample (NIS) represents a diverse population in terms of geography, race, and ethnicity, facilitating the applicability of our findings to COVID-19 patients with AMI covered under both private insurance and Medicare Advantage plans, as well as Medicaid plans. As our study relies on the NIS, there are several associated limitations that can be addressed in future studies. The 2021 NIS does not have data on the vaccination status of the patients, which could influence outcomes. In addition, the NIS does not include details about their medication history and treatment plans during their hospitalization. Our study could not include critical physical examination, laboratory test results, and radiographic findings that could help categorize and study the patients based on severity. Mistakes in codes and inputs at physician level may also influence our results. One needs to also acknowledge that race and ethnicity are highly heterogeneous, and the categories employed in this analysis may not fully capture this diversity [16,61].

Finally, while our study found various fluctuations in the incidence of AMI among COVID-19 cases, as lockdown protocols were heavily State-dependent, and the NIS does not contain State-related data, it is vital to encourage additional retrospective studies which can help understand how lockdowns impacted the incidence as well as the quality of care and outcomes of such patients [62,63,64].

## 5. Conclusions

Our retrospective study, via one of the biggest hospitalization records, has shown that the incidence of AMI among COVID-19 patients between April 2020 and July 2021 was highest in January 2021, and from July 2021, the numbers rose constantly until December to reach a new peak. The identification of specific periods of heightened vulnerability, such as the observed peaks in AMI incidence, can inform targeted interventions and resource allocation strategies to better manage cardiovascular complications. We also observed differences in the comorbidities and characteristics of COVID-19-positive adults with AMI between the two years, with patients experiencing more complications in 2021, and a higher adjusted odds ratio of mortality. This study stresses the need for research endeavors to address the changing impact of the pandemic on cardiovascular health. It also emphasizes the importance of collaboration among healthcare providers, researchers, and policymakers to develop comprehensive strategies for reducing the cardiovascular disease burden during the pandemic.

## Figures and Tables

**Table 1:** AMI cases among adult COVID-19 patients between April 2020 and December 2021 in the United States.

Time	Total Number of AMI Cases	Total Number of COVID-19 Cases	AMI Events among COVID-19 Cases
Apr-20	4525	123,925	3.65%
May-20	2145	60,865	3.52%
Jun-20	1775	59,035	3.01%
Jul-20	3545	118,725	2.99%
Aug-20	2515	75,970	3.31%
Sep-20	1785	55,635	3.21%
Oct-20	3130	98,525	3.18%
Nov-20	7265	207,120	3.51%
Dec-20	8050	220,060	3.66%
Jan-21	10,520	261,279	4.03%
Feb-21	3790	103,270	3.67%
Mar-21	2445	75,705	3.23%
Apr-21	2725	93,035	2.93%
May-21	1765	55,215	3.20%
Jun-21	715	26,825	2.67%
Jul-21	2005	79,275	2.53%
Aug-21	7055	227,059	3.11%
Sep-21	6645	181,289	3.67%
Oct-21	4410	111,135	3.97%
Nov-21	4865	112,515	4.32%
Dec-21	8505	195,530	4.35%
Overall	90,180	2,541,992	3.55%

**Table 2:** Characteristics of COVID-19 patients with AMI in 2020 vs. 2021 in the United States.

Variable	AMI among COVID-19 Patients in 2020 (*n* = 34,735) (%)	AMI among COVID-19 Patients in 2021 (*n* = 55,445) (%)	*p*-Value
Mean age (±SD)	72.38 (13.31)	70.31 (13.71)	<0.01
Weekend admission	26.8	26.7	0.766
Female	39.3	40.5	<0.01
Primary payer			<0.01
Medicare	69.9	66.1	
Medicaid	8.7	9.4	
Private	15.5	17.8	
Race			<0.01
White	55.6	65.0	
Black	17.8	16.1	
Hispanic	18.0	12.4	
Median household income			<0.01
0–25th percentile	36.8	36.8	
26th to 50th percentile (median)	29.6	27.4	
51st to 75th percentile	20.5	21.7	
76th to 100th percentile	13.1	14.1	
Hospital characteristics			
Hospital bed size			<0.01
Small	23.5	24.7	
Medium	29.9	30.7	
Large	46.6	44.6	
Location/Teaching status			<0.01
Rural	10.9	13.1	
Urban non-teaching	18.0	18.5	
Urban teaching	71.2	68.3	
Region of hospital			<0.01
Northeast	18.7	16.6	
Midwest	2.1	22.9	
South	38.7	42.8	
West	16.5	17.7	
Comorbidities			
Sarcoidosis	0.3	0.3	0.791
SLE	0.6	0.5	0.495
Rheumatoid arthritis	2.1	2.2	0.290
Hyperthyroidism	0.6	0.6	0.615
Hypothyroidism	14.1	13.2	<0.01
Hypertension	27.9	28.1	0.543
Dyslipidemia	53.2	49.4	<0.01
Smoking	28.1	30.1	<0.01
Diabetes	49.0	45.9	<0.01
CKD	40.5	39.2	<0.01
Prior CABG	8.4	7.6	<0.01
Prior PCI	10.7	9.2	<0.01
Family history of CAD	4.5	4.0	<0.01
Peripheral vascular disease	4.4	4.3	0.604
Prior stroke	8.3	7.6	<0.01
Cirrhosis	5.2	5.8	<0.01
Alcohol abuse	1.8	2.4	<0.01
Prior MI	9.4	8.8	<0.01
Obesity	20.4	25.5	<0.01
Drug abuse	2.0	2.8	<0.01
COPD	20.7	21.9	<0.01
Mean CCI Score	5.04 (3.35)	4.91 (3.36)	<0.01

**Table 3:** Mortality rates among AMI events in COVID-19 patients between April 2020 and December 2021 in the United States.

Time	Mortality of AMI Patients with COVID-19
Apr-20	43.20%
May-20	31.90%
Jun-20	33.20%
Jul-20	31.60%
Aug-20	31.20%
Sep-20	32.50%
Oct-20	31.20%
Nov-20	28.50%
Dec-20	31.90%
Jan-21	32.50%
Feb-21	26.40%
Mar-21	27.20%
Apr-21	26.40%
May-21	30.90%
Jun-21	23.10%
Jul-21	34.40%
Aug-21	35.60%
Sep-21	35.30%
Oct-21	33.90%
Nov-21	34.50%
Dec-21	34.20%
Overall	32.70%

**Table 4:** Arrhythmias, procedures, and complications among COVID-19 patients with AMI in 2021 vs. 2020 (reference).

Cardiac Arrhythmias				
Variable	*p*-Value	aOR	Lower 95% CI	Upper 95% CI
Atrial fibrillation	0.424	1.013	0.982	1.045
Supraventricular tachycardia	0.527	1.023	0.954	1.096
Ventricular tachycardia	<0.001	1.126	1.062	1.194
Complications				
PCI	<0.001	1.627	1.454	1.822
Cardiogenic shock	0.841	1.008	0.931	1.092
IABP	0.565	0.902	0.636	1.281
AKI	<0.001	1.078	1.047	1.110
AIS	<0.001	1.215	1.113	1.328
Cardiac arrest	<0.001	1.106	1.050	1.166
Invasive mechanical ventilation	<0.001	1.133	1.096	1.172
Died	0.043	1.032	1.001	1.064

## Data Availability

The authors are not allowed to share the database. However, anyone interested in the database can contact the HCUP https://hcup-us.ahrq.gov/nisoverview.jsp (accessed on 1 February 2024).
